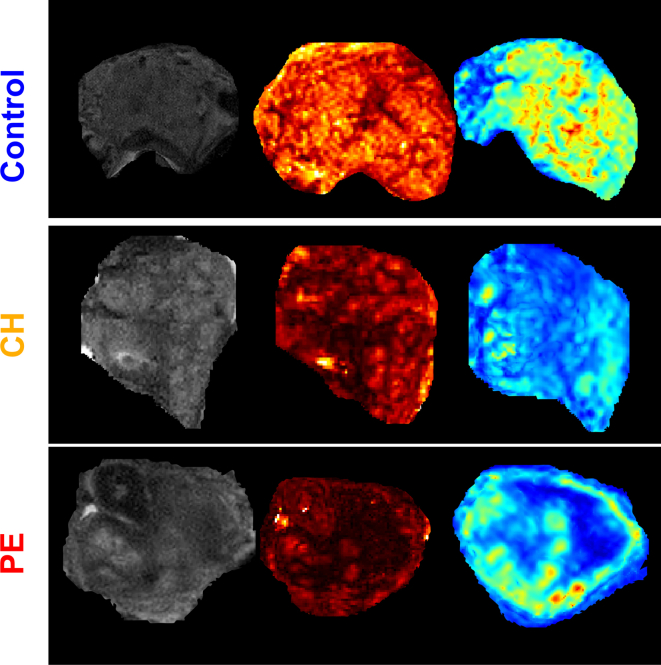# Cardiac and placental imaging (CARP) in pregnancy to assess aetiology of preeclampsia

**DOI:** 10.1016/j.placenta.2022.03.012

**Published:** 2022-05

**Authors:** Kathleen Colford, Anthony N. Price, Julie Sigurdardottir, Anastasia Fotaki, Johannes Steinweg, Lisa Story, Alison Ho, Lucy C. Chappell, Joseph V. Hajnal, Mary Rutherford, Kuberan Pushparajah, Pablo Lamata, Jana Hutter

**Affiliations:** a Centre for Medical Engineering, King's College London, London, UK; b Centre for the Developing Brain, King's College London, London, UK; c Department of Congenital Heart Disease, Evelina Children's Hospital, London, United Kingdom; d Academic Women's Health Department, King's College London, London, UK

**Keywords:** Pregnancy, Preeclampsia, Placenta, MRI, CMRI, Quantitative MRI, CVD

## Abstract

**Introduction:**

The CARP study aims to investigate placental function, cardiac function and fetal growth comprehensively during pregnancy, a time of maximal cardiac stress, to work towards disentangling the complex cardiac and placental interactions presenting in the aetiology of pre-eclampsia as well as predicting maternal Cardiovascular Disease (CVD) risk in later life.

**Background:**

The involvement of the cardiovascular system in pre-eclampsia, one of the most serious complications of pregnancy, is evident. While the manifestations of pre-eclampsia during pregnancy (high blood pressure, multi-organ disease, and placental dysfunction) resolve after delivery, a lifelong elevated CVD risk remains.

**Method:**

An assessment including both cardiac and placental Magnetic Resonance Imaging (MRI) optimised for use in pregnancy and bespoke to the expected changes was developed. Simultaneous structural and functional MRI data from the placenta, the heart and the fetus were obtained in a total of 32 pregnant women (gestational ages from 18.1 to 37.5 weeks), including uncomplicated pregnancies and five cases with early onset pre-eclampsia.

**Results:**

The achieved comprehensive MR acquisition was able to demonstrate a phenotype associated with pre-eclampsia linking both placental and cardiac factors, reduced mean T2* (p < 0.005), increased heterogeneity (p < 0.005) and a trend towards an increase in cardiac work, larger average mass (109.4 vs 93.65 gr), wall thickness (7.0 vs 6.4 mm), blood pool volume (135.7 vs 127.48 mL) and mass to volume ratio (0.82 vs 0.75). The cardiac output in the controls was, controlling for gestational age, positively correlated with placental volume (p < 0.05).

**Discussion:**

The CARP study constitutes the first joint assessment of functional and structural properties of the cardiac system and the placenta during pregnancy. Early indications of cardiac remodelling in pre-eclampsia were demonstrated paving the way for larger studies.

## Highlights

• A comprehensive assessment of both cardiac and placental function during pregnancy.
• Step towards understanding the intertwined cardiac and placental pathways in PE.
• Pregnancy as a unique window of observation into pathophysiology and future cardiovascular risk.
• Placental parameters deviate significantly between PE and uncomplicated pregnancies.
• Early indications for a remodelling of the heart during PE are demonstrated.

## 1 Introduction

Pre-eclampsia is one of the most common and serious complications of pregnancy with an incidence of 3–7% [1]. Pre-eclampsia is a multi-organ disease with potential sequelae including renal and liver dysfunction, coagulopathy, pulmonary oedema and eclampsia (seizures) for the mother and growth restriction and premature birth for the fetus, usually related to the need for planned delivery for maternal or fetal indications. While the substantial pregnancy-related morbidity and mortality risk mostly ceases shortly after delivery, pre-eclampsia carries a lifelong increased risk of cardiovascular disease (CVD) for the woman [[2], [3], [4]].

### 1.1 Placental and cardiac changes associated with pre-eclampsia

Placental histopathological evidence such as maternal malperfusion, chronic villitis and increased fibrin deposition [5] has been linked to insufficient transformation of the spiral arteries in early pregnancy [6]. Placental assessment during pregnancy is hampered by difficulties in isolating the feto-placental compartment. Magnetic Resonance Imaging (MRI) was recently able to identify highly distinctive signatures of placentas from women with pre-eclampsia [7] and chronic hypertension [8]. This includes decreased mean diffusivity, indicative of changes in the placental microstructure such as the density of the villous trees [9,10] and reduced T2*, related to placental oxygenation via the paramagnetic properties of deoxygenated haemoglobin.

The maternal cardiac physiology is altered in pregnancy to meet the demands of a growing fetus and profound maternal hemodynamic changes occur. Pre-eclampsia (PE) results in additional cardiac changes, including a lower or larger cardiac output depending on early or late PE [11], abnormal cardiac geometry, diastolic dysfunction [4], increased vascular resistance and left ventricular mass [12] as well as myocardial oedema [13].

### 1.2 Combined studies

Much of the placental and maternal cardiac data has been gathered and interpreted in isolation and research studies are often focused on system-level biomarkers or purely on changes observed in the placenta. The relative contribution of prior risk factors and newly acquired injury from pre-eclampsia cannot currently easily be disentangled. Combined studies may constitute a first step towards this effort by providing additional knowledge on the pathway of events.

The CARdiac and Placental (CARP) study aims to contribute to closing this gap by assessing both the placenta and the maternal heart comprehensively and simultaneously during the time of maximal stress, in pregnancy. It aims to provide an opportunity to investigate both the aetiology of pre-eclampsia and, potentially at a later stage, to predict maternal CVD risk in later life, opening opportunities for targeted screening and treatment via:

- developing, for the first time, a safe in-pregnancy a robust combined MRI protocol covering the maternal cardiovascular system, placenta and fetus.
- phenotyping cardiac and placental function and any correlation between the two.
- Adding to the evidence from ultrasound studies by providing additional functional and structural insights for early cardiac pathophysiology associated with pre-eclampsia using this novel imaging capacity in combination with a collection of clinical information.

## 2 Methods

### 2.1 Study design

This prospective observational cohort study was undertaken at Guy's and St Thomas' NHS Hospital, London. All women gave written informed consent as part of the ethically approved CARP study (London Dulwich Ethics Committee 08/LO/1958). Exclusion criteria included contraindications to MRI due to metallic implants, pace-makers, claustrophobia, body-mass-index (BMI) > 40 kg/m2, inability to give informed consent, diagnosis of a prior structural cardiac pathology, age under 16 or over 50, multifetal pregnancy or diagnosis of any fetal anomaly.

Prospectively specified data collection included baseline demographic characteristics, maternal, and neonatal outcomes.

Participants with uncomplicated pregnancies, pre-eclampsia and chronic hypertension were recruited, but the final assignment of the cohort was performed after the outcomes were obtained. Women were then classified as ‘controls’ when the following criteria were fulfilled: no diagnosis of a hypertensive disorder at enrollment and until delivery, no significant past medical history, no pregnancy complications (including gestational diabetes mellitus), delivery at term with birth weight between the 3rd and 97th centile (calculated using International Fetal and Newborn Growth Consortium for the 21st Century version 1.3.5), thus excluding potential obstetric confounders of placental change.

Pre-eclampsia was prospectively defined using the international consensus definition [14]: gestational hypertension accompanied by one or more of the following new-onset conditions at or after 20 weeks’ gestation: proteinuria, acute kidney injury, liver involvement, neurological complications, hematological complications, and uteroplacental dysfunction. Preeclampsia superimposed on chronic hypertension was defined as the additional presence of maternal organ dysfunction consistent with preeclampsia. Women with a diagnosis of chronic hypertension before pregnancy but no diagnosis of pre-eclampsia were assigned to the chronic hypertension cohort. Women not matching these criteria were considered separately. Women with comorbid pre-eclampsia and diabetes were excluded to not confound the effects. All women diagnosed with PE were on medication at the time of scan. Four on labetalol, four on nifedipine and one on other antihypertensive agents (methyldopa, doxazosin) or a combination of these.

No formal sample size was calculated for power of outcome variables as this was an exploratory study describing a novel technology development.

### 2.2 Examination

All participants were scanned on a clinical 1.5T Philips Ingenia scanner in maternal supine position [15] using a combined posterior and torso (dStream) coil. Maternal comfort in the supine position was maximised by elevating the woman's head and legs, as well as splitting the scan in two 30-min sessions with a rest break in-between [15]. Continuous monitoring of electrocardiography, blood pressure and oxygen saturations with frequent verbal checks were maintained throughout the scan. Between 4 and 12 blood pressure readings were recorded before and during the scan by a trained midwife using an appropriately sized cuff. Maternal body temperature was recorded using an in-ear thermometer before and after each session.

The two sessions focussed respectively on placental sequences and cardiac sequences, with the order randomised by study ID to avoid bias by minimising any effect of stress caused by uncertainty or anxiety that may be felt in the initial stage of scanning [16].

The protocol is graphically illustrated in Fig. 1 and all scanning parameters are given in Table 1, the considered cohorts are shown in supporting Fig. S1.

**Fig. 1:**
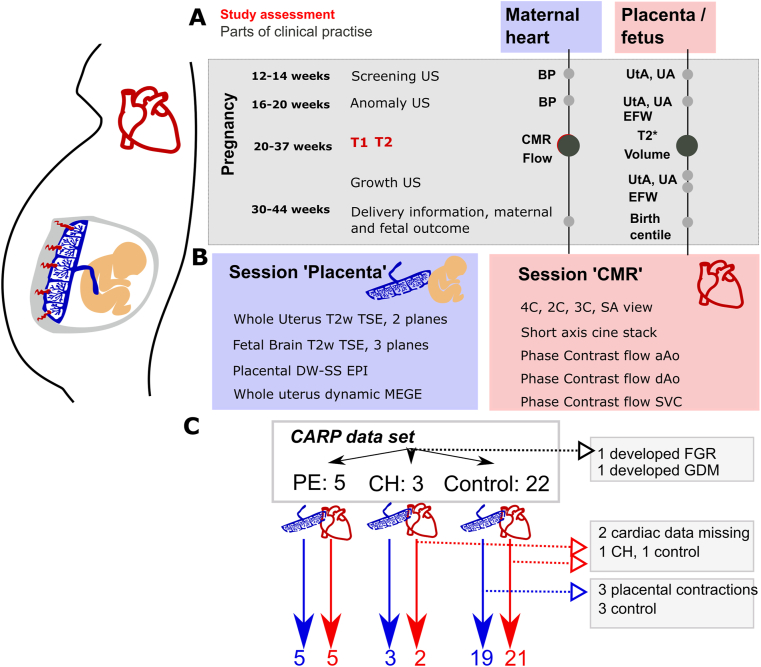
Graphical illustration of the CARP study. (A) Time course illustrating the quantitative evaluations performed for the heart, placenta and to assess the fetal growth. In Grey clinical examinations and in red the time point of the extra MRI scan as part of CARP. (B) Illustration of the two sessions, which are performed in random order to avoid bias. (C) Patient flow diagram illustrating the different groups. The results for the Chronic Hypertensive group will be included but not analysed in detail due to the group size. (For interpretation of the references to color in this figure legend, the reader is referred to the Web version of this article.)

**Table 1:** Scanning parameters.

Session ‘Placenta’	
Anatomical	**T2-weighted Turbo-Spin Echo**
	TR = 30s, TE = 180 ms,Matrix size = 384 x 384 x 150, Resolution 1.25 x 1.25 × 2.5 mm
T2* - static	**Multi-Echo Gradient Echo**
	TR = 23s, TE = 12.599 ms/65.34 ms/118.08 ms/170.821 ms/223.562 ms, Dynamics = 2, Matrix size = 224 x 224 x 850, Resolution = 2.5 x 2.5 × 2.5 mm
Diffusion	**Pulsed Gradient Echo Spin Echo, Echo Planar Imaging**
	TR = 6.6s, TE = 78 ms, Matrix = 512 x 512 x 56, Resolution = 2 x 2 × 4 mm, b = 375 (6 directions), b = 750 (6 directions)
T2* - dynamic	**Multi-Echo Gradient Echo**
	TR = 6.4s, TE = 7.84 ms/60.574 ms/113.308 ms/166.041 ms, Dynamics = 30, Matrix size = 256 x 256 x 360, Resolution = 2.5 x 2.5 × 2.5 mm
**Session ‘CMR’**	
Cine bSSFP	**bSSFP**
	30 phases, FA = 60, TR = 4 ms, TE = 2 ms, Resolution = 1.7 x 1.7 × 8 mm, ∼11sec breath-hold
Short axis stack	**bSSFP**
	TR = 4.1s, TE = 2 ms, 14 slices, sense = 2, resolution: 2x2x10mm
Phase Contrast (BH option)	30 phases, 14 s breath-hold, resolution = 2.5x2.5 × 8mm, sense = 2
Phase Contrast (free breathing)	30 phases, resolution = 2.5x2.5 × 8mm, sense = 2, NSA = 4

### 2.3 CMR

The cardiac session consisted of anatomical and functional Cardiac MRI sequences. These were optimised for safe use during pregnancy by minimising heating (measured in Specific Absorption Rate) and acoustic noise of the scanner. A dedicated microphone system (OptoAcoustics) was used to measure the acoustic noise of all sequences in a stable environment, an empty bore at fixed position to ensure it did not exceed 97 db (A).

Cine balanced Steady State Free Precession (bSSFP) sequences were acquired, using retrospective ECG gating, in standard planes (4-chamber, 2-chamber and 3-chamber view, short axis, aortic outflow), with 30 cardiac phases (∼11sec breath-hold, FA = 60, TE/TR∼2/4 ms, resolution: 1.7x1.7 × 8mm) and a short-axis stack (with 14 slices, sense = 2, resolution: 2x2x10mm). Examples are given in supporting Fig. S1. Phase contrast flow sequences (30 phases, 14 s breath-hold, resolution: 2.5x2.5 × 8mm, sense = 2) at the ascending Aorta (AA), descending Aorta (DA) and superior vena cava (SVC) were acquired with individually adapted encoding velocities. The planning of these locations for the flow measurements is shown exemplary in supporting Fig. S2.

Anatomy of the left ventricle (LV) was studied from the end diastolic frame of the Cine bSSFP short axis stack. Manual segmentation of the LV myocardium was performed, and 3D meshes built using our computational anatomy toolkit [17]. A statistical shape model was then built with a Principal Component Analysis (PCA), finding the main modes of anatomical variation as described in previous studies [18]. The impact of PE is assessed by an optimised linear discriminant analysis of the first 12 modes as described in Ref. [19].

Cardiac output (CO) was computed using the cine short axis stack: stroke volume (end diastolic volume minus end systolic volume) multiplied by the heart rate. Cardiac work (CW) was computed using the mean arterial pressure.

Cardiac output (CO) = Stroke volume x heart rate [L/beat]

Mean Arterial Pressure (MAP) = Diastolic BP + 1/3 (Systolic BP – Diastolic BP) [mmHg]

Cardiac work (CW) = Stroke volume x Mean Arterial Pressure (MAP) x heart rate [L x mmHg / min]

### 2.4 Placental and fetal imaging

The placental sequence session consisted of whole uterus anatomical scans and dedicated placental acquisitions. Fetal brain and whole uterus structural imaging was performed using 2D-single-shot Turbo-Spin-Echo (resolution 1.5x1.5 × 2.5 mm, FOV = 320x320 × 110mm, TE = 180 ms) [21] in five planes, two opposing sagittal oblique, a straight transverse plane centered on the fetal brain and two additional planes, covering the entire uterus in two stacks coronal and sagittal to the maternal habitus. These images were used in the current study for clinical reporting only.

Functional placental MRI acquisition includes T2* relaxometry [[22], [23], [24], [25]] and Diffusion MRI [9,10], based on previous projects [9,26]. Therefore, a multi-echo gradient-echo sequence (2.5 mm isotropic, FOV = 300x300 × 110mm, TEs = 11,58,117,176 ms) is used to obtain the data for T2* mapping and a twice-repeated diffusion-weighted single-shot EPI sequence with parameters adapted to the expected diffusivity (1 b = 0, 6 b = 375 and 6 b = 750), (TR = 6.6s, TE = 78 ms, Matrix = 512 x 512 x 56, Resolution = 2 x 2 × 4 mm) is acquired to get information sensitive on the microstructure of the placenta. Both are repeated twice to improve robustness against subclinical contractions.

The placental parenchyma was manually outlined on the first functional scan and propagated to the following scans. T2* maps were obtained using monoexponential fitting. Mean placental T2* as well as histogram skewness and kurtosis as measures of heterogeneity were calculated. Mean diffusivity (Apparent diffusion coefficient ADC) was calculated.

### 2.5 Analysis

Several evaluations were performed on the data. First, the evolution of the assessed cardiac (mean systolic BP, mean diastolic BP, CO and CW) and placental (mean T2*, placental volume from T2*, kurtosis, skewness, mean ADC) markers against gestational age were analysed for the controls by correlation. In order to interrogate on the relationship between heart and placenta measures in these controls we performed partial correlations (spearman) adjusted for gestational age at scan. Then, in a next step, the potential of the methods described here was assessed comparing the available pre-eclampsia cases against controls. Due to the five pre-eclamptic cases being scanned significantly later than controls (median [IQR] 34.2 [32.7–35.3] week vs 28.7 [22.7–34.0] weeks, p = 0.0075), they were matched with controls 2:1 on nearest gestational age only and thereafter compared using the Wilcoxon rank-sum test which are presented with boxplots. We also present a correlation matrix where all controls and preeclampsia cases are included. It reports the Spearman rho correlation coefficients between heart and placenta factors, stratified by cohort and for the whole study sample.

### 2.6 Results

32 scans were obtained with the CARP protocol, two scans were in the same woman at different time points. Partial data including at least the structural assessment of placenta and heart was obtained in participants. Complete combined datasets were obtained from 27 out of 32 performed combined scans from 31 participants: The cardiac assessment could not be completed in one woman due to claustrophobia and one due to increasing BP. The placental assessment in seven participants was hampered by subclinical contractions. However, due to the described strategy to repeat the functional acquisitions, data without contractions was available in all but three of the cases.

We considered the available data from all 32 scans; one was excluded as the woman developed fetal growth restriction and one as the woman developed GDM. Of the remaining datasets, five were diagnosed with pre-eclampsia and four with chronic hypertension (of which cardiac data are available in three). The chronic hypertensive group was added into the plots in this section for completeness but no further results will be shown due to the small group size. The cohort characteristics for CARP are reported in Table 2. The flow of patients is depicted graphically in Fig. 1C.

**Table 2:** Study cohort characteristics.

	Health Control	Pre-eclamptic
**Age [years], mean (IQR)**	33.59	34.16
**Age [years], range**	25–44	26–40
**BSA [km2], median (IQR)**	1.84	1.93
**Gestational age at the time of scan [weeks], median (IQR)**	29.1	32.05
**Gestational age at time of scan [weeks], range**	18.1–37.5	29.2–36.6
**Gestational age at birth, mean**	39.386143	34.78472
**Gestational age at birth, range**	37.1–40.85714	33.28571–37.42262
**Birth weight [grams], mean**	3330.294118	1848.333333
**Birth weight [grams], range**	2700–3655	1310–3070
**Previous Preeclampsia**	0	1
**Sitting blood pressure (MRI) [Systolic/Diastolic, mmHg, median IQR]**	110.25	135.75
**Total Supine blood pressure (day of MRI) [Systolic/Diastolic mmHG, median, IQR]**	104.65	128.57
**Start of scan Supine blood pressure (day of MRI) [Systolic/Diastolic mmHG, median, IQR]**	102.75	130.25
**After break Supine blood pressure (day of MRI) [Systolic/Diastolic mmHG, median, IQR]**	111.76	127.75

### 2.7 Cardiac results

The CMR protocol was successfully adapted for safe use in pregnancy while maintaining image quality as can be observed in Fig. 2, illustrating 2Ch, 4Ch and SA views in end-systole and end-diastole. The blood pressure recordings over the entire scan time in Fig. 2 show a clear separation between women with and without pre-eclampsia (c and d) with similar progress between the ordering of the two protocol components “Placenta” and “CMR” (a and b). The later was assessed by comparing the difference between the second BP measurement correlating with the start of the MRI scan in both halves.

**Fig. 2:**
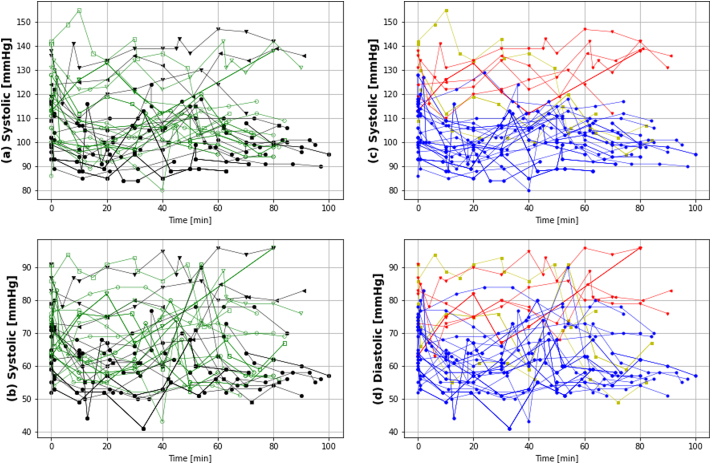
The blood pressure (diastolic and systolic) results over the entire MRI scan including the blood pressure taken before starting the scan is illustrated. Thereby, the green lines with open symbols in (a) and (b) correspond to the cases acquired in order “Placenta”->“CMR” and the black lines with filled symbols “CMR”->“Placenta”. (c) and (d) are color coded by cohort with red corresponding to the pre-eclampsia cases and blue the control cases. The chronic hypertensive group was added in yellow for completeness but no further analysis has been done due to the small group size. (For interpretation of the references to color in this figure legend, the reader is referred to the Web version of this article.)

**Fig. 3:**
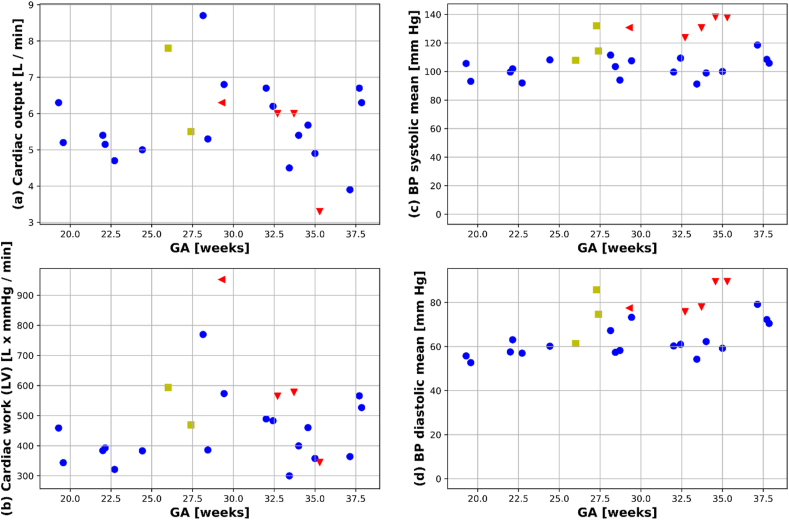
Quantitative cardiac indices. (a) Cardiac Output (CO) calculated from the short axis stack. (b) Cardiac work (CW) computed using CO and the mean arterial pressure. (c) Mean systolic blood pressure averaged over the scan. (d) Mean diastolic blood pressure averaged over the scan. Healthy controls are marked with blue dots and pre-eclamptic subjects with red triangles. The chronic hypertensive group was added with yellow squares for completeness but no further analysis has been done due to the small group size. (For interpretation of the references to color in this figure legend, the reader is referred to the Web version of this article.)

In the 21 healthy controls with complete cardiac data, gestational age was associated with an increase in diastolic blood pressure (r = 0.468, p < 0.05) but not in systolic blood pressure (r = 0.255, p > 0.1). The calculated CW (SV x BP) and CO calculated from the phase contrast flow data are given in Fig. 3.

**Fig. 4:**
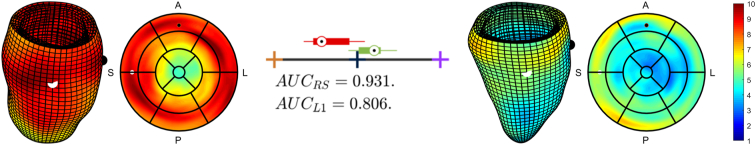
Signature of pre-clampsia (PE) in the left ventricle found by the linear discriminant analysis of the 12 first modes of the statistical shape model. The resulting “PE mode” is illustrated by its extreme (i.e. at ± 3 std) shapes and thickness bullseye plots, and with the box-plot distributions in between them: PE subjects (red box-plot) have thicker walls and more spherical apical regions than the control pregnancies (green box-plot). This is a preliminary finding, illustrated by the large gap between the resubstitution (RS) and cross-validation (Leave 1 out or L1) area under the curve (AUC) of the discriminant analysis. (For interpretation of the references to color in this figure legend, the reader is referred to the Web version of this article.)

In the matched sample analysis, early onset pre-eclamptic women had higher average systolic (p < 0.005) and diastolic (p < 0.005) BP than control participants. No difference in CO was found, the CW was trending towards higher values in the women with early onset pre-eclampsia but did not reach significance due to the size of the cohort.Analysis of the 3D LV shape reveals an anatomical mode that differentiated pre-eclampsia to control subjects (p < 0.01), and its visual assessment shows that women with early onset pre-eclampsia had thicker walls and a more spherical apex (See Fig. 4). Individual analysis of LV dimensions (mass, length, blood pool volume) based on 3D reconstructed anatomies does not lead to any significant differences, but to a tendency of PE subjects to show larger average mass (109.4 vs 93.65 gr), wall thickness (7.0 vs 6.4 mm) blood pool volume (135.7 vs 127.48 mL), and mass to volume ratio (0.82 vs 0.75).

### 2.8 Placental results

#### 2.8.1 Control cases against GA

**Fig. 5:**
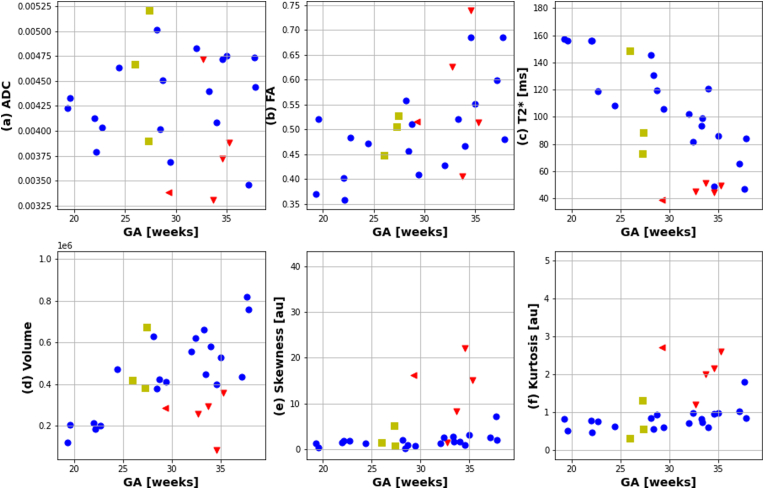
Quantitative placentas results are depicted evaluated for the entire placental parenchyma for the entire considered population (CARP and non-CARP subjects). (a) Mean ADC in [mm2/s] and (b) Mean FA [au] from the diffusion data. (c) Mean T2* in [ms], (d) volume in [mm3], (e) kurtosis [au] and (f) skewness[au] of the histogram distribution from the T2* data is shown. Healthy controls are marked with blue dots and the pre-eclamptic group with red triangles. The chronic hypertensive group was added with yellow squares for completeness but no further analysis has been done due to the small group size. [au] = arbitrary unit. (For interpretation of the references to color in this figure legend, the reader is referred to the Web version of this article.)

The placental results are visually displayed for the anatomical, T2* and diffusion scans in supporting Fig. 3 for one case in each cohort at a similar gestational age of between 32 and 34 weeks and quantitatively over gestational age in Fig. 5. In the uncomplicated pregnancies, placental mean T2* decreased significantly from on average 160 ms at 20 weeks' gestation to 80 ms at 35 weeks’ gestation. Placental volume increased from ∼200 cm3 at 20 weeks to ∼800 cm3 at 40 weeks. ADC decreased slightly and FA increased from ∼0.36 at 20 to ∼0.7 at 40 weeks. The skewness and kurtosis increased. Regression of the control measures against gestational age was performed and showed significant association for the placental measures with T2*(p < 0.005), Volume(p < 0.005), Skewness (p < 0.005) and Kurtosis (p < 0.005).

#### 2.8.2 PE results

**Fig. 6:**
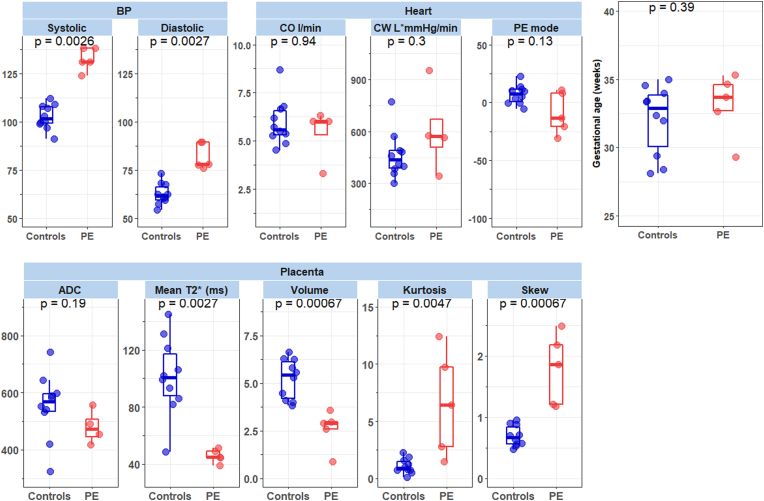
The five PE cases were each matched to 2 controls by nearest gestational age at scan. We confirm expected blood pressure differences and present exploratory group comparisons on markers of MRI-derived heart and placental parameters in boxplots. The p-values were obtained using the Wilcoxon Rank Sum test. PE mode was obtained from the PCA analysis of the mesh model analysis and volume. ADC = Apparent Diffusion Coefficient.

To assess the results in the pre-eclampsia cohort, the age-matched sample was used and significantly decreased mean T2* (p < 0.005), decreased mean Apparent Diffusion Coefficient (p > 0.1) and significantly increased heterogeneity, assessed using skewness (p < 0.005) and kurtosis (p < 0.005) compared with controls was shown (Fig. 6).

#### 2.8.3 Correlations between cardiac and placental results

**Fig. 7:**
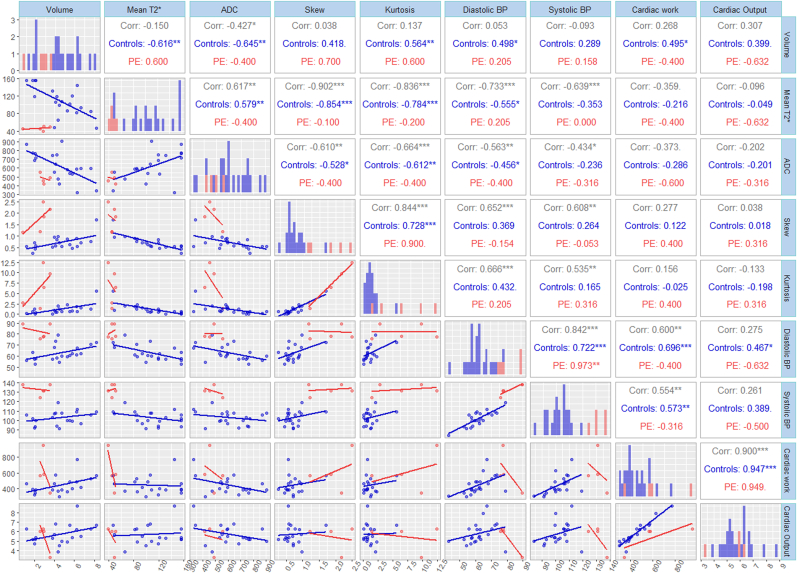
Correlation matrix stratified by participant cohort (controls/PE). Mean T2*, ADC (apparent diffusion coefficient), Volume, Skew and Kurtosis refers to placental MRI-derived metrics. The upper triangle provides the spearman rho correlation coefficients (p-values <0.05, <0.01 and < 0.001 are represented by *, ** and *** respectively). Coefficients in grey are on the whole sample, in blue are controls (n = 23) and red are pre-eclampsia cases (n = 5). The diagonal are histograms for the variables included.”. (For interpretation of the references to color in this figure legend, the reader is referred to the Web version of this article.)

The correlation between the cardiac and placental results is depicted visually for all measures in a matrix analysis in Fig. 7. We have further analysed the correlations between cardiac and placental results controlling for GA, these results show that CO is positively correlated with placental volume T2* (p < 0.05) in the control group. CW was correlated with systolic pressure (p < 0.05) but CI was not correlated with systolic and diastolic pressure in the control group.

## 3 Discussion & conclusion

### 3.1 Principal findings

A comprehensive and safe-in-pregnancy MRI protocol to visualise and quantify placental function and cardiovascular activity in 60 min was demonstrated for the first time in a cohort of uncomplicated pregnancies and pregnancies complicated by pre-eclampsia. 27 of 32 participants had successfully acquired cardiac and placental data during pregnancy with the proposed protocol corresponding to an over 85% success rate. Demonstrated correlations of the quantitative cardiac and placental measures with gestational age in the control cases are an important step towards understanding normal development. First correlations link early onset pre-eclampsia to increased CW at the time of the MRI and show correlations between cardiac and placental factors, setting the scene for gaining insight into the intertwined contributions of cardiac and placental pathology.

### 3.2 Discussion of the results

The CARP study provides for the first time the ability to observe correlations between cardiac and placental markers obtained at the same time during pregnancy. The individual components of placental function assessment and cardiac quantification, however, can be related to previous research done in isolation on either the heart or placenta. The observed placental phenotype of early onset pre-eclampsia with a decrease in T2* and an increase in heterogeneity was in line with similar studies [7,22,27]. The illustrated spread of results in the chronic hypertension cohort, shown previously [8] was confirmed in the CARP data but not analysed as a cohort here due to the limited numbers. The observed changes agree well with similar results shown in a cohort of women with fetal growth restriction and placental insufficiency [22,28].

The cardiac results in the healthy cohort showed the expected stable behaviour of cardiac work (CW) during pregnancy, but they did not show the expected progressive increase in cardiac output (CO) as pregnancy advances [[29], [30], [31]]. The mean CO of 5.8 L/min was lower than the 6.4 L/min reported to be characteristic at 28–32 weeks of gestation [32]. Accordingly, the cardiac work (CW) was also lower in our controls (median of 399.5 vs. the 524 mmHg*L/min reported at 28–32 weeks of gestation [32]).

The initial analysis demonstrates that PE subjects, when compared to controls, had a significantly higher blood pressure during the scan, no increase in CO and the tendency to increase in CW. Our early PE subjects have the descriptors of CO and BMI (median 6.0 L/min and 26.2 kg/m^2^ respectively) approximately in the borderline that separates early to late PE, where late PE subjects are reported to display larger CO and BMI [11]. Our initial analysis also reveals wall thickening and increased sphericity in the study of the 3D left ventricle anatomy, what was in line with the changes observed in women with pre-eclampsia postpartum [4] as well as with echo results during pregnancy [33] and an initial study using CMR in pregnancy [13]. Left ventricular thickening independent of changes in diameter and length has also been characterised as a chronic phenotype caused by the hypertensive insult during pregnancy [35].

On the methodological side, since results reveal CO values that are lower in controls and larger in early PE compared to literature, there is no clear bias potentially caused using a different imaging modality (MRI in this study vs. echocardiography in the literature). Besides, no indexed values of CO are provided because previous reports of a low resistance-high output pattern at term might be in part due to classification of CO relative to the nonpregnant standard [34].

Anecdotally, the one late pre-eclamptic case in the cohort, who was excluded from the analysis due to her GDM, was diagnosed at 35 weeks and delivered at term, showed functional placental measures in line with the other pre-eclamptic cases (low T2*, high skewness) but had the highest placental volume and lowest cardiac work result among the pre-eclamptic cohort. This could be a very early indication of a differently emerging phenotype of these late-onset pre-eclamptic cases, resulting in similar placental insufficiency but with fewer cardiac consequences.

### 3.3 Clinical implications

Fetal MRI is used in clinical practice for selected indications (such as further evaluation of fetal neurological abnormalities). However, there are constraints to widespread adoption due to the cost and expertise required and is thus not well suited as a screening modality. It can, however, be of great added value in high-risk groups. This study constitutes only a first step towards gaining knowledge allowing to explore the predictive power of a combined assessment which can later inform and be translated to other modalities or tests.

The observed cardiac differences could be a first indication of an adaptive concentric remodelling response to the increased pressure in the pre-eclamptic participants. This could potentially aid in the explanation of an increased risk of cardiovascular disease despite apparently similar levels of cardiac functionality in this group. This study constitutes a first exploratory analysis and feasibility assessment. To assess the potential for clinical employment, comparison with state-of-the art biomarker assessment such as Placental Growth Factor must be conducted.

For the aim of predicting later life CVD, close clinical follow-up after the index pregnancy, with potentially a repeat CMR scan is required, but was not included in this study.

Finally, further focus on groups with significant risk for pre-eclampsia such as chronic hypertension (pooled risk 16%, 12.6–19.7%), previous pre-eclampsia (8.4%, 7.1–9.9%), and pre-pregnancy body mass index (BMI) > 30 (7.1%, 6.1–8.2%) among others [36,37] could be of further clinical interest.

### 3.4 Research implications

The developed assessment of placenta and maternal heart during pregnancy is a promising tool for future research into the intertwined effects of pre-eclampsia on both organs. Future studies enrolling larger cohorts of women with hypertensive pregnancies to obtain distinct phenotypes of early and late onset preeclampsia, potentially linked with changes in later CVD risk, can potentially help further elucidate the cascade of events preceding pre-eclampsia.

The present study was performed supine, both to improve the conditions for imaging such as proximity to the surface coils and to increase consistency and comparability by eliminating differences in the tilt angle. The maternal position is, however, known to influence the cardiac measures such as stroke volume and cardiac output, with a potential 16.4% reduction in cardiac output shown in the supine position compared to the left lateral position [30,38]. These results from a reduced flow in the IVC, shown to correlate with gestational age, degree of the tilt and previous position [15].

This choice might, in line with previous studies, result in changes in perfusion and oxygenation which needs to be taken into account when comparing absolute values. To allow this in the future, we also measured the flow in the inferior vena cava (IVC) similar to Hughes et al. [15] using the phase contrast protocol as stated above. This was performed in the “placenta” half of the protocol due to the better coil coverage for the IVC. The obtained comprehensive information on the maternal cardiac system can furthermore potentially help to assess these changes in more detail.

### 3.5 Strengths and limitations

One key strength of this study is the achieved comprehensive assessment of both cardiac and placental health during pregnancy. The developed MRI examination provides the ability to collect robustly quantitative insights which are, especially for placental function, unique and not obtainable other ways due to the isolated nature of the feto-placental compartment. This study thereby looks beyond placental structure. Diffusion MRI is sensitive to the motion of water within the placenta and hence able to inform on the microstructure such as the density of the villous trees [9,10]. T2* relaxometry exploits the paramagnetic properties of deoxygenated haemoglobin [[22], [23], [24], [25]] to allow indirect insights into placental oxygenation. Similarly, Phase Contrast flow measurements were included to obtain functional insights. The achieved comprehensive coverage of both cardiac function and placental function and the shown ability to achieve this robustly in more than 90% of the participants paves the way for future studies in this area.

Maternal comfort, safety robustness and hence the resulting ability to obtain these data sets even in patients with significant clinical disease have been shown in this study and are essential for any future investigation.

The establishment of a safe and well monitored examination to be conducted in a consistent maternal supine pose as described above, is a further strength of this study as only such a consistent setup allows robust quantification of cardiac and placental function.

A limitation is the low number of participants especially in the pre-eclamptic cohort. This is reflective of the often short time scales between diagnosis of pre-eclampsia and delivery. But the achieved successful demonstration of both technical feasibility of such a combined examination and first indication of an emerging phenotype relating heart and placenta in women with early onset pre-eclampsia is an essential step towards such larger studies.

A further limitation is the lack of matched echocardiography data. Such comparisons in larger studies would significantly strengthen the work.

### 3.6 Perspectives

Our proof-of-concept study and the demonstrated comprehensive data collection during pregnancy fills an important gap in experiments. Structural and functional cardiac and placental MRI data acquired during pregnancy demonstrate a placental and cardiac phenotype in preeclampsia that can contribute to understanding the complex mechanisms underlying the aetiology of placental and cardiac dysfunction in preeclampsia. Further studies to determine whether there is a potential role for predicting the subsequent development of cardiovascular disease after the index pregnancy in high-risk groups (eg, those with chronic hypertension, gestational hypertension) would be of interest. The study thus has the potential to identify a unique window of opportunity to aid clinical management decisions, measuring effectiveness of new therapies and future potential intervention. Longer term, the data created from this study can potentially inform both screening for onset of pre-eclampsia and later cardiovascular life risk for women at high risk. These screening efforts might be inspired by the created new insights which can potentially translate to more widely available modalities and tools.

## Figures and Tables

**figs1:**
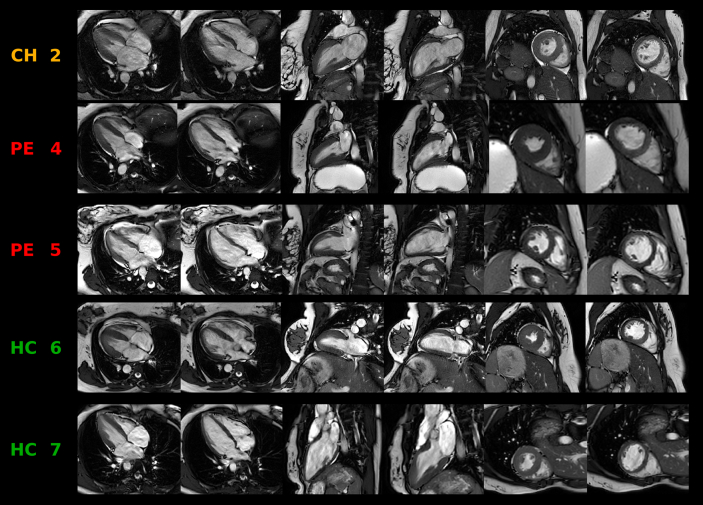


**figs2:**
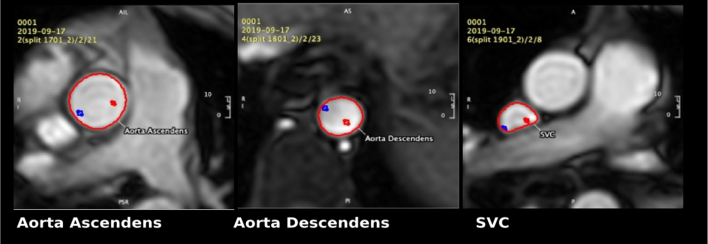


**figs3:**